# Psychometric properties of the Persian version of the celiac disease adherence test questionnaire

**DOI:** 10.1186/s12876-020-01396-8

**Published:** 2020-07-29

**Authors:** Zeinab Nikniaz, Mohammad Asghari Jafarabadi, Saeideh Ghaffarifar, Zahra Saeedi, Zahra Akbari Namvar, Masood Shirmohammadi

**Affiliations:** 1 Liver and gastrointestinal diseases research center, Tabriz University of medical sciences, Tabriz, Iran; 2 Road Traffic Injury Research Center, Department of Epidemiology and Biostatistics, Faculty of Health, Tabriz University of Medical Sciences, Tabriz, Iran; 3 Medical Education Research Centre, Health Management and Safety Promotion Research Institute, Tabriz University of Medical Sciences, Tabriz, Iran; 4 Student research committee, Tabriz University of medical sciences, Tabriz, Iran

**Keywords:** Celiac disease, Gluten-free diet, Adherence, CDAT, Validation, Persian

## Abstract

**Background:**

A gluten-free diet (GFD) is the only effective treatment for celiac patients and assessing adherence to this diet is important. Celiac disease Adherence Test (CDAT) is a valid English-language questionnaire that is used for assessing the adherence to the GFD. In the present study, we aimed to translate the CDAT questionnaire in Persian and evaluate its validity and reliability.

**Methods:**

In the present cross-sectional study, CDAT was translated and back-translated by three bilingual professional translators. Content validity was evaluated by 12 gastroenterologists and nutritionists. To assess the construct validity, 230 patients with celiac disease were randomly selected from the national celiac disease registry database. Internal consistency of the items and test-retest reliability were assessed by Cronbach’s alpha and Intraclass Correlation Coefficient (ICC). To assess the convergent validity of the questionnaire, the correlation coefficient between the CDAT score and anti-tissue transglutaminase immunoglobulin A (anti-t-TG-IgA) titer was assessed.

**Results:**

The content validity index, content validity ratio, and impact score of the Persian version of CDAT (Pv-CDAT) were 0.97, 0.95, and 4.61 respectively. Three significant factors were extracted and according to the confirmatory factor analysis the three-factor model had adequate fitness (chi-square *p*-value of 0.74, root mean error of approximation: 0.001 [95% CI: 0.001–0.083], comparative fit index: 1, standardized root mean squared residual: 0.04, and coefficient of determination: 0.78). The questionnaire had good feasibility with the floor effect of 3.1% and the ceiling effect of 0.4%. Moreover, it has high internal consistency (Cronbach-alpha: 0.71) and test-retest reliability (ICC: 0.78). The correlations between CDAT categories and anti-tTG-IgA categories showed a significant correlation between the two tests (*r* = 0.53; *P* < 0.001).

**Conclusions:**

The results of the present study showed that the Pv-CDAT questionnaire with high validity, reliability, and internal consistency can be used for assessing adherence to the GFD in adult celiac patients in Iran.

## Background

Celiac disease (CD) is an autoimmune enteropathy that is associated with gluten intolerance in people with a genetic background [1]. In these patients, even the use of a small amount of gluten protein can damage small intestinal mucosa [2] that is associated with different gastrointestinal and non-gastrointestinal clinical manifestations [3, 4]. A gluten-free diet (GFD) is the only effective treatment for these patients [5, 6] and strict compliance to this diet could reverse the symptoms and improve nutritional status [7, 8]. The result of a systematic review showed that adherence to the GFD was low and it ranged from 42 to 91% depending on the definition and method of assessment [9]. Assessing adherence to a GFD is methodologically challenging. Different methods such as clinician evaluation, serology tests, or endoscopy with duodenal biopsy histology are used to define the level of non-adherence to the GFD [10]. However, these tests have limitations such as inadequate accuracy, low sensitivity, high cost, and invasiveness. Leffler et al. developed a Celiac Dietary Adherence Test (CDAT) which is the fast, and sensitive method of assessing the adherence to the GFD [11]. This questionnaire has seven items, easy to administer and the validity and reliability of the English version were confirmed [11]. From then, it was used in different studies [12–14] and it has been translated into the Spanish language with appropriate psychometric properties [15].

To the best of our knowledge, CDAT has not been translated to Persian yet and clinician evaluation and serology tests are used in physician’s daily practice. However, the accuracy and sensitivity of these tests are questionable. Hence, considering the approved validity of the CDAT questionnaire for use in the evaluation of adherence to GFD [11], in the present study, it was aimed to translate the CDAT questionnaire in Persian and evaluate its validity and reliability.

## Methods

The present explorative cross-sectional study was done from July 2018 to January 2020.

The translation and adaptation were done using the Forward-Backward translation method. For this, at first, the permission of the author of CDAT, Dr. D. A Leffler was taken by Email. Then two translators who were fluent in English independently translated the questionnaire into Persian (forward translation). The two versions were compared and any discrepancies were resolved by discussion and a final translated questionnaire was created. This version of the translated questionnaire was back-translated to English by an independent native English language speaker who was unaware of the English version. This version of the questionnaire was sent to Dr. D. A Leffler to confirm.

### Statistical analysis

We analyzed the data using the IBM© SPSS© Statistics version 20 (IBM© Corp., Armonk, NY, USA) and STATA version 14.

### Assessment of face validity

Face validity was assessed by the panel of experts (12 experts) and oral feedback of 15 celiac patients immediately after the completion of the questionnaire. The experts were asked to provide their opinion about the importance of each question on the five-point Likert scale. The impact score (IS) was calculated and the values greater than 1.5 were considered acceptable. According to patients and experts opinion, only the minor change in one item was made.

### Assessment of content validity

The Persian version of the CDAT questionnaire (Pv-CDAT) (Table S1) was tested for content validity in a group of 12 gastroenterologists and nutritionists. The experts were asked to complete a form to evaluate the simplicity, clarity, relevance, and necessity of each question. After the minor change in one item, the final Persian version of the questionnaire was made. Further, we computed the Content validation ratio (CVR) for each item based on the Lawshe method with the participation of 12 experts. The minimum acceptable level of CVR was 0.56. The content validity index (CVI) was also calculated for each item and the minimum acceptable level of CVI was 0.79.

### Assessment of construct validity

The construct validity of the questionnaire was assessed through Exploratory Factor Analysis (EFA) and Confirmatory Factor Analysis (CFA).

A total of 230 celiac patients were selected from the celiac disease registry database in East Azerbaijan. The patients were selected randomly using a computer-generated random number. EFA was conducted on 130 patients, and CFA was performed on 100 patients. The inclusion criteria were: age 18–65 years and registration in East Azerbaijan celiac disease registry database.

The patients were asked to complete the Pv-CDAT questionnaire. This questionnaire has 7 questions on the five Likert scale type. The scores are summed to obtain a final score (from 7 to 35). A score of less than 13 indicates good adherence, 13–17 a moderate adherence, and higher than 17 a poor GFD adherence [11].

Kaiser–Meyer–Olkin (KMO) test and Bartlett’s test of sphericity were evaluated to confirm sample adequacy for elicitation of the factors. Principal axis analysis followed by a varimax rotation was used to test the factor constructs of all the seven items.

CFA was conducted to assess the goodness of fit between a hypothesized model provided in Leffler et al. study and the data obtained from 100 celiac patients in the present study who did not participate in the EFA. Chi-square, root mean error of approximation (RMSEA), comparative fit index (CFI), normed fit index (NFI), Tucker-Lewis index (TLI), and standardized root mean squared residual (SRMR) were measured. The acceptable values were Chi-square *p*-value> 0.05, RMSEA< 0.08, TLI & NFI > 0.9, SRMR < 0.08.

### Assessment of convergent validity

For assessing convergent validity, the level of anti-tissue transglutaminase immunoglobulin A (anti-t-TG-IgA) was measured as a serological test of GFD adherence using AESKULISA® tTG new generation kit (code:3503; Wendelsheim Germany). According to Kit instruction, the levels of less than 12 U/ml were considered good adherence, 12–18 U/ml a moderate adherence, and > 18 U/ml a poor adherence. The correlation between the adherence level obtained by Pv-CDAT and IgA anti-tTG was assessed by the Kendall-tau correlation test.

### Assessment of the reliability

The internal consistency was measured through Cronbach’s alpha. Besides, the ceiling and flooring effect is measured and values lower than 20% were considered acceptable. For calculating internal consistency, the mean scores of 130 questionnaires were used.

Test-retest reliability was assessed on 26 random samples of patients who completed the questionnaire 2 weeks apart. The Intraclass correlation coefficient (ICC) was used for analysis.

## Results

In the present study, 230 celiac patients participated in the psychometric study of the Pv-CDAT scale. The mean age of participants was 36.36 ± 12.90 years and 61.7% of them were female. The mean disease duration was 4.26 ± 4.89 years. Other baseline characteristics of the participants are provided in Table 1.

**Table 1:** Baseline characteristics of celiac patients who participated in psychometrics properties assessment (*n* = 230)

Variable	Mean ± SD
**Age (years)**	36.36 ± 12.90
**Disease duration (Years)**	4.26 ± 4.89
**Frequency (%)**	
**Sex (M:F)**	88 (38.26)/142 (61.73)
**Married**	166 (72.17)
**Positive family history of celiac**	14 (6.08)
**Education level**	
**Illiterate**	64 (27.82)
**High school**	159 (69.13)
**University**	7 (3.01)
**Anti-t-TG-IgA**	
**< 12**	95 (41.3)
**12–18**	73 (31.7)
**> 18**	62 (26.9)

*Anti-t-TG-IgA* Anti-tissue-transglutaminase-IgA

The CVI, CVR, and impact score of the Pv-CDAT were 0.97, 0.95, and 4.61 respectively (Table 2).

**Table 2:** The results for the content and face validity of Persian version of CDAT questionnaire

Items	CVI*	CVR**	IS***
**Have you been bothered by low energy level during the past 4 weeks?**	1	1	4.90
**Have you been bothered by headaches during the past 4 weeks?**	0.97	0.82	4.20
**I am able to follow a GFD when dining outside my home**	1	1	4.90
**Before I do something I carefully consider the consequences**	0.93	0.82	4.18
**I do not consider myself a failure**	0.90	1	4.09
**How important to your health are accidental gluten exposures?**	1	1	5
**Over the past 4 weeks, how many times have you eaten foods containing gluten on purpose?**	1	1	5
**The mean of whole questionnaire**	0.97	0.95	4.61

*CVI* Content validity index, *CVR* Content validity ratio, *IS* Impact score, *GFD* Gluten-free diet

*values > 0.56 are acceptable; ** values > 0.79 are acceptable; *** values> 1.5 are acceptable

As shown in Table 3, using factor analysis, three significant factors were extracted that account for 61% of the variance. The results of the KMO and Barlett sphericity test showed the model adequacy (KMO: 0.70 and Bartlett’s test of sphericity *p*-value< 0.001). The first factor included questions 1 and 2, the second factor included questions 3, 4, and 5 and the third factor included questions 6 and 7.

**Table 3:** Results of Exploratory Factor analysis of Pv-CDAT (*n* = 130)

Items	Factor 1	Factor 2	Factor 3
**Have you been bothered by low energy level during the past 4 weeks?**	0.67		
**Have you been bothered by headaches during the past 4 weeks?**	0.55		
**I am able to follow a GFD when dining outside my home**		0.63	
**Before I do something I carefully consider the consequences**		0.42	
**I do not consider myself a failure**		0.40	
**How important to your health are accidental gluten exposures?**			0.45
**Over the past 4 weeks, how many times have you eaten foods containing gluten on purpose?**			0.43

Extraction Method: Principal Axis Factoring. Rotation Method: Varimax with Kaiser Normalization

**Fig. 1:**
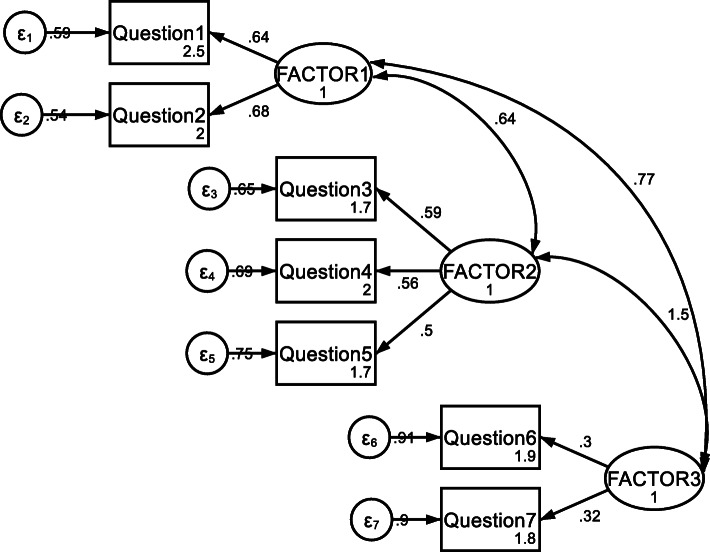
Results of Confirmatory Factor analysis of the Pv-CDAT (*n* = 100)

Figure 1 shows the results of the confirmatory factor analysis. According to these results, the three-factor model had acceptable fitness (Chi-square *p*-value: 0.74, RMSEA: 0.001 [95% CI: 0.001–0.083], CFI: 1, SRMR: 0.04, and coefficient of determination: 0.78).

At the stage of determining convergent validity, the correlations between CDAT categories and anti-tTG categories was statistically significant (*r* = 0.53; *P* < 0.001).

The Floor and ceiling effects were 3.1 and 0.4% respectively. The Cronbach alpha and ICC of the questionnaire were 0.71 and 0.78 [0.31–0.89].

## Discussion

CDAT questionnaire is easy to use, fast, and valid tool for evaluating the adherence to the GFD [11]. Previously, two other questionnaires including the Biagi score (four questions) [16] and the theory of planned behavior (TPB) questionnaire (19 questions) [17] were developed for assessing GFD adherence in celiac patients. Considering that the CDAT with a limited number of questions provided the widest evaluation and it is used frequently in different research, we selected this questionnaire for translation [15]. Leffler et al. showed that it has higher sensitivity and specificity compared with serological tests in assessing GFD compliance. In this study, we translated this questionnaire into Persian and evaluated the psychometric properties of it in celiac patients. According to the results, the Pv-CDAT had good content validity, internal consistency, and test-retest reliability. Previously, only the psychometric properties of the English [11] and Spanish [15] versions had been studied. The Spanish version of the questionnaire had moderate internal consistency [15]. The internal consistency of the final English questionnaire was not assessed. However, the pre-final questionnaire had high internal consistency (Cronbach α: 0.80) [11]. Besides, we showed high test-retest reliability of the Persian version which is in accordance with the results of the original English version (Pearson r: 0.82) [11].

The Pv-CDAT had CVI of 0.97 and all questions had the CVI equal or higher than 0.9. All questions had acceptable CVI according to the Lawshe Table (CVI > 0.56). In addition, CVR of the Pv-CDAT was 0.95 and all questions had CVR ≥ 0.82. The impact score of the Pv-CDAT was 4.61 and all questions had IS higher than the minimum acceptable score [18].

Considering the results of factor analysis, the Pv-CDAT comprised three factors that confirm the structure of the English version both in the number of factors and their combination. This supports the construct validity of this questionnaire. The Spanish version of the questionnaire also fitted well with those originally proposed by Leffer et al. [15]. Considering that in both versions, Spanish and Persian, the number of items kept similar to that of the original questionnaire, the good construct validity is expected.

We also checked the correlation between the results of the CDAT and anti-tTG-IgA test and showed that these two tests were highly correlated. Previously, Leffler et al. have reported a high correlation between CDAT score and anti-tTG-IgA titer [11]. This correlation was not studied in the Spanish version of CDAT [15]. Although anti-tTG-IgA was not the best measurement to assess the adherence to GFD [19], it is routinely used for this purpose.

The results of this study should be interpreted considering the limitation of the present study. We just tested the questionnaire on the East Azerbaijan population, this may normally restrict the generalization of its results. Besides, we assessed convergent validity by analyzing the correlation between the score of the CDAT questionnaire and anti-tTG-IgA. As mentioned before, this test is not the gold standard to check GFD adherence. So, it is suggested that future studies consider the patients in different populations and also evaluated the correlation between the Pv-CDAT and other tests such as expert evaluation of adherence.

## Conclusions

In conclusion, the results of the present study showed that the Pv-CDAT questionnaire had high validity, reliability, and internal consistency. Considering that compared with other methods, this questionnaire is easier to use, it can be applied for assessment of the level of adherence to GFD in adult celiac patients in Iran. In addition, in the pandemic situation like COVID-19, the questionnaires can be used to “tele-monitor” patients.

## Supplementary information

**Additional file 1: Table S1.** The Persian version of the CDAT (Pv-CDAT) questionnaire.

## Data Availability

The datasets supporting the conclusions of this research are included in the article.

## Abbreviations

CDAT: Celiac disease adherence test
Pv-CDAT: Persian version of celiac disease adherence test
CVR: Content validity ratio
CVI: Content validity index
IS: Impact score
GFD: Gluten-free diet
CD: Celiac disease
Anti-t-TG-IgA: Anti-tissue transglutaminase immunoglobulin A
RMSEA: Root mean squared error of approximation
CFI: Comparative fit index
SRMR: Standardized root mean squared residual
