# Supplementary material for: Psychometric properties of the Persian version of the celiac disease adherence test questionnaire
Source: BMC Gastroenterol. 2020 Jul 29;20:247. doi: 10.1186/s12876-020-01396-8 (PMC7389158; doi:10.1186/s12876-020-01396-8)
Supplement: Supplementary file 1 — Additional file 1: Table S1. The Persian version of the CDAT (Pv-CDAT) questionnaire. [file 12876_2020_1396_MOESM1_ESM.docx]

**Table S1: the Persian version of the CDAT (Pv-CDAT) questionnaire**

| **سوال** | **۱** | **۲** | **۳** | **۴** | **۵** |
| --- | --- | --- | --- | --- | --- |
| **آیا طی ۴ هفته گذشته، از سطح پایین انرژی رنج برده اید؟** | هرگز | خیلی کم | گاهی اوقات | اغلب | همیشه |
| **آیا طی ۴ هفته گذشته، از سردرد رنج برده اید؟** | هرگز | خیلی کم | گاهی اوقات | اغلب | همیشه |
| **من می­توانم از رژیم غذآیی فاقد گلوتن، در خارج از خانه پیروی کنم.** | کاملا موافقم | موافقم | نظری ندارم | مخالفم | کاملا مخالفم |
| **قبل از اینکه کاری را انجام دهم، به دقت نتایج آن را مدنظر قرار میدهم** | کاملا موافقم | موافقم | نظری ندارم | مخالفم | کاملا مخالفم |
| **من خودم را یک فرد شکست خورده نمیدانم.** | کاملا موافقم | موافقم | نظری ندارم | مخالفم | کاملا مخالفم |
| **تا چه حد مصرف اتفاقی گلوتن برای سلامتی شما مهم است؟** | بسیار مهم | تاحدی مهم | خنثی/مطمئن نیستم | اهمیت کم | اهمیتی ندارد |
| **در طی ۴ هفته گذشته، چند نوبت به عمد غذای حاوی گلوتن را مصرف کرده اید؟** | ۰(هرگز) | ۱-۲ | ۳-۵ | ۶-۱۰ | بیشتر از ۱۰ |
